# Amino Acids Transport and Metabolism 2.0

**DOI:** 10.3390/ijms21041212

**Published:** 2020-02-12

**Authors:** Mariafrancesca Scalise, Cesare Indiveri

**Affiliations:** Department DiBEST (Biologia, Ecologia, Scienze della Terra), Unit of Biochemistry and Molecular Biotechnology, University of Calabria, Via P. Bucci 4C, 87036 Arcavacata di Rende, Italy; mariafrancesca.scalise@unical.it

## Abstract

This editorial aims to summarize the 19 scientific papers that contributed to this Special Issue.

The nutritional status and, hence, the well-being of all living organisms are dramatically influenced by amino acid availability. Indeed, besides being the building blocks of proteins, amino acids represent fuel and signalling molecules and are, therefore, *conditio sine qua non* for life maintenance. The adequate amino acid distribution and homeostasis are guaranteed by an equilibrium among the absorption, the intracellular metabolism, and the transmembrane traffic. The concerted regulation of all these processes is a very complex issue since each of the twenty proteinogenic amino acids has a different concentration that needs to be maintained constant inside and outside the cells [1,2]. This reflects the different abundance of each amino acid in proteins. The concentration and the distribution are also linked to the role of some special amino acids; this is the case for glutamine, a conditionally essential amino acid that, under conditions of increased proliferation rate, is required in a greater amount to fulfil energy needs. In humans, the amino acid homeostasis is even more intriguing since it is strictly linked to diet, and changes in diet follow habits that are associated with different human populations and, in some cases, fashion. These aspects have, therefore, great relevance to human nutrition and health. The average plasmatic concentration of the amino acids in human cells ranges from 3 µM of the least represented, i.e., aspartate, to 500 µM of the most abundant, i.e., glutamine [3]. To achieve the strict control of the homeostasis, cells harbour an intricate network of proteins with enzymatic or transport activity that mediates the utilization of amino acids and their traffic across the plasma and intracellular membranes. The transmembrane flux is mediated by membrane transporters that, therefore, are major players in this network. Transporters for amino acids have peculiar features, such as a wide specificity, i.e., the same protein can recognize more than one amino acid. Moreover, amino acid transporters exhibit diverse transport mechanisms to allow either a massive uptake of each amino acid from the cell exterior or exchange of different amino acids among cells for harmonizing the intracellular amino acid pool. While the study of enzymes is at a more advanced stage, the knowledge and the systematic classification of membrane transporters are still incomplete due to the delay in the availability of appropriate methodologies for handling these hydrophobic proteins. However, over the years, a lot of novel information has been obtained on membrane transporters, using different and complementary experimental tools from in vivo to in vitro systems, as well as in silico methodologies. The increasing number of recently solved 3D structures of amino acid transporters boosted investigations on molecular mechanisms and structure/function relationships. At present, there are transporters whose structure and function is well understood, and many others are still at an initial knowledge state or are completely unknown. Therefore, the investigation of different aspects of each transporter is still very active and contributes to a significant advancement in understanding the mechanisms of control of amino acid homeostasis. However, a large gap still has to be filled to disclose the intricate world of amino acid homeostasis. The aim of this Special Issue, that contains 13 original articles and 6 reviews, is drawing the *status artis* on some key aspects of membrane transporters and their connection with metabolism. In particular: (i) relevant basic knowledge on membrane transporters, still *in nuce*, are furnished; (ii) novel information on regulatory aspects of well-characterized membrane transporters are provided; (iii) findings on applied research that are relevant to human health are given. Indeed, the link between amino acid transporter defects and inherited metabolic diseases is nowadays clearly assessed and several molecular mechanisms through which the defects in transporter function cause the disorders, are already well known. Regarding the first aspect (i), the article by Tripathi et al. deals with the deorphanization of the membrane transporter SLC38A10 that is located in the ER and the Golgi apparatus and that is responsible for glutamine transport. The authors suggest that SNAT10 is a transceptor, i.e., a transporter with a receptor function, as SLC38A2 and SLC38A9 [4]. Among novel transporters, the article by Shi et al. reports the functional characterization of two amino acid transporters specific for arginine and citrulline. The importance of this finding resides in the first description of membrane transporters in watermelon fruits where they play an important role in fruit development [5]. Another newly identified plasma membrane transporter is the AlaE from *E. coli* that is responsible for the efflux of L-alanine. The role of this transporter should be that of preventing the toxic accumulation of alanine when the bacteria grow in a peptide-rich environment, such as in an animal intestine [6]. An interesting finding has been reported by Vigorito et al. regarding a novel role of the non-proteogenic amino acid lanthionine, a by-product of H_2_S metabolism. A role for lanthionine has been described in causing the alteration of calcium homeostasis. This pathogenic role allowed for the classification of lanthionine as a novel uremic toxin [7]. Regarding the second aspect (ii), the article by Zaitsev et al. describes a weak anticonvulsant effect exerted by the antibiotic ceftriaxone through the rat glutamate transporter EAAT2 of the plasma membrane, opening perspectives for the explanation of brain functioning [8]. In the same section, Convertini et al. identifies the sites of interaction for two transcription factors of the human mitochondrial aspartate/glutamate carrier (AGC2); the work is conducted by employing different up to date techniques for promoter identification, and the role of AGC2 regulation in tumours has been described. The article by Dabrowska et al. deals with another transcription factor responsible for regulating the expression of the plasma membrane transporter SNAT3, one of the eleven members of the SLC38 family. In particular, the transcription factor Sp1 modulates the expression of SNAT3 following ammonia treatment on mice astrocytes. Interestingly, the effect of Sp1 is mediated by the phosphorylation levels of protein kinase C [9]. A peculiar regulatory mechanism is that described in the article by Arthur et al. In this study, the peroxynitrite that is produced during chronic inflammation has been found to inhibit the sodium-dependent alanine and glutamine transporters in brush border membranes; these findings highlight a specific function of peroxynitrite and amino acid transporters in inflammatory processes [10]. The last article of this section describes interesting regulatory aspects for the vacuolar arginine transporter of *S.lycopersicum* (tomato). The transporter is revealed to be osmoregulated and cation-sensitive, responding to physiological changes of the vacuolar compartment. Furthermore, the positive regulation of the arginine transport activity by cholesterol has been shown for the first time [11]. As highlighted above, the third section (iii) of this Special Issue includes articles and reviews describing the relevance of membrane transporters for amino acids to human health. This issue is firstly reviewed by Yahyaoui et al., who reports a general and broad description of the involvement of amino acid transporters in human metabolic disorders. The review focuses on the importance of the diagnostic characterization of the defects, at the molecular level, as a starting point for designing appropriate therapies [12]. Following the same scope, the group of excitatory amino acids (EAATs) belonging to the SLC1 family has been taken into consideration by several authors, reflecting the importance of these proteins in human physiopathology. EAATs have been reviewed in terms of their involvement in neurotransmission as well as being key players in the glutamate metabolism by Magi et al. [13]. The same proteins have been evaluated in the frame of disorders of the central nervous system by reviewing the molecular mechanism derangement causing the pathological states. Interestingly, the unexpected role of the EAAT transporters in participating in the redox homeostasis has been highlighted as an additional link with the human pathology [14]. Another family of amino acid transporters very relevant to human health is the SLC7; indeed, several authors addressed key open questions regarding these proteins. Hafliger et al. has reviewed the role of the fifth member of the SLC7 family, LAT1 (SLC7A5), as an eminent target for drugs due to its wide over-expression in human cancers [15]; in the same frame, Singh et al. proposes novel and potent inhibitors of LAT1, employing a combined strategy of virtual screening of a one million molecule library with in vitro testing of the top-ranked hits [16]; the seventh member of the SLC7 family, SLC7A7, has been studied by Bodoy et al., who propose building a mouse model KO for this arginine plasma membrane transporter as a promising tool to study the molecular mechanisms underlying the lysinuric protein intolerance [17]. Finally, another interesting finding is the cryo-EM 3D map of the complex LAT2 (SLC7A8)-4F2hc that has been solved at 13 Å resolution, employing a functionally over-expressed recombinant protein [18]; the availability of an improved 3D structure of LAT2 opens an important perspective in the field of drug design for human therapy given the role of this protein in several human diseases. This Special Issue also includes articles on mitochondrial carriers linked to human diseases with a wide range of severity. As an example, the review by Monnè et al. focuses on the role played, in energy production, by mitochondrial carriers involved in the transport of amino acids; notably, the defects of these carriers, caused by inherited mutations, are responsible for a series of human diseases [19]. The article by Petralla et al. describes the link between AGC1 (aspartate/glutamate carrier 1) deficiency with neurological disorders by using in vitro and in vivo mouse disease models [20]. Lastly, the article by Traustason et al. deals with the employment of computational strategies for the optimization of the amino acid composition of cell growth media. This research field is gaining attention due to the applications to cell lines used in the production of biopharmaceutical drugs [21]. In conclusion, this Special Issue on amino acid transport and metabolism is well placed in the wider framework of membrane transporter research. Indeed, these proteins represent one of the most important gambles in the future of worldwide research for their relevance in unveiling fundamental processes in all living organisms as well as in the industrial applications.
